# Massey’s Electricity in the Diseases of Women

**Published:** 1889-08

**Authors:** 


### *MASSEY'S ELECTRICITY IN THE DISEASES OF WOMEN.*

*Editors Buffalo Medical and Surgical Journal:*

GENTLEMEN—In a criticism of my work on "Electricity in the Diseases of Women," which was made the basis of an editorial in the April number of the JOURNAL, certain statements appear which clearly demand attention at my hands, and you will confer a favor upon me by granting space for a reply, as well as for an expression of my thanks, now extended, for the commendatory portions of the review.

Is it presumptuous, on my part, to hint that the comprehensive editorial "we" shields in this instance the shoulders of a young and enthusiastic manipulator of the knife? Not to mention external and accidental evidences, it is more than apparent that the criticism on which the editorial is based, was not written by one practically conversant with electric currents, and we may safely take it as the first critique on the work yet appearing from the hands of an ultra-surgical extremist; and from this point of view it deserves particular consideration. As the production of an opponent of all electrical applications in gynecology, it is somewhat inconsistent that the unqualified condemnation expressed in the last paragraph should be preceded by the seductive statement on the first page:

"The want of such a treatise has long been felt by the general practitioner, and to this class of the profession it should be practically valuable."

Now, as to the particular sins of this voluminous but largely misguided critic, I must complain first and most emphatically of the charge of dishonesty contained on page 549, in which the insinuation is made that only picked cases showing good results are given. A disinterested reader of the work would certainly arrive at a different conclusion, and even the critic's complaint of some poor results reported, shows the impartial nature of the clinical reports.

Among other evidences of the critic's total want of comprehension of currents and their powers, is the remarkable statement he makes that meters are unnecessary. He quotes the author as saying that it is "criminal to use a galvanic current (through insensitive mucous membranes) without adequate means of knowing the amount actually passing through the patient," and proceeds to disagree with this statement, alleging that by the author's admissions the sensations of the patient furnish the real indications. Would the critic banish scales from the drug-store merely because patients differ in susceptibility to drugs? Is there no use in knowing how much quinine or opium is given when we can watch for cinchonism or narcotism? If we are to take sensations as a guide, let the critic give us a standard scale of sensations. The writer's experience is that the comparison of such psychological conditions is extremely difficult. He has seen the same evidences of pain from fifteen milliampères in one woman as were produced by 350 in another. Surely such arguments are more than puerile. Their animus is apparent further on when the critic dwells with complacency on the fact that electricity gives pain as well as the knife. Strong currents do hurt some at times, it is true, but whether too much for the results gained, may be safely left to the judgment of the patient. The complaints of the surgeons about them are somewhat amusing.

The critic objects to the statement that an interchange of particles occurs in the interpolar regions. Is he not aware that the passage of particles through the body from pole to pole may be demonstrated? The facts of electrolysis are a proven portion of pure physics.

Among the clinical reports contained in the work, he is apparently more at home, but his painstaking analysis of the cases would do him greater credit if he were not so apparently anxious to misunderstand and misjudge the author's claims at every step. In spite of the prominence given to the following sentence in the preface: "It should be stated also that the author does not wish to assume the position of recommending the routine use of any one agent or procedure, to the exclusion of other rational remedies in the medical or surgical treatment of any single class of diseases. It was merely in the interest of clearness and accuracy that many of the cases mentioned in these pages were confined to electrical applications alone," he charges "a disposition to claim any and everything in the most reckless manner," although inadvertently admitting further on that some cases reported simply show a slight improvement and are still under treatment. The cases were reported as encountered; and as the author was not then examining into the virtues of hot water, ergot, purgatives, etc., he confined his attention to the agent under consideration. The results were largely satisfactory to both the physician and patient, and it is hoped that their faithful, though somewhat tedious, portrayal in the work may be of benefit to the profession. The remote history of each case, so far as obtainable, will be given in a future edition.

Assuming again the role of an electrical expert, the critic intimates that the author must be unfamiliar with Apostoli's more recent writings, when acute inflammatory affections, particularly perimetritis, are still cited as absolute contra-indications to the use of *strong currents*. That the currents mentioned by Apostoli are *faradic*, seems to have escaped him.

But, to mention but another item in this elaborate attempt at picking flaws, the reviewer quotes, in connection with the subject of extra-uterine pregnancy, the author's statement that after electrical feticide "two courses are open to the surgeon, either laparatomy for the removal of the dead mass, which is now more easily performed, or a mere promotion of the efforts of nature in removing the mass by absorption," adding, "if electricians are going to advocate laparatomy to remove the dead mass, the profession will no longer pay any attention to their inconsistent protests against also removing the living mass." In this he differs widely from no less a surgical authority than Gaillard Thomas, who says (*Amer. Sys. Gyn.*, Vol. II., p. 191,):

" Unless the imminence of rupture render feticidal efforts hazardous and delay for this purpose inadvisable, the life of the fetus should always be destroyed prior to fetal viability before laparatomy is resorted to. After fetal death, from the very instant that it is accomplished, diminished vascularity rapidly establishes itself, and every day, every hour, renders the chances of a subsequent operation better."

The reviewer is wrong, too, in setting up the necessity of a diagnosis as a pre-requisite to electrical feticide. Currents, such as advised, may be employed at the first suspicion of this unfortunate condition, and will do no harm whatever to the mother if the suspicion is without foundation.

I trust, Messrs. Editors, that after this exposition of the groundless nature of these complaints, you will permit me to avail myself of this opportunity to call attention to an important omission from the pages of this little work, pointed out by another reviewer, Dr. Franklin H. Martin, of Chicago. In giving directions for using the incandescent electric-light current of the Edison variety, I failed to mention, what was then unknown to me, that the Westinghouse Company have a number of *incandescent* installations in various portions of the country in which an alternating current is used. Such currents are, of course, thoroughly inadmissible for this purpose, and may be dangerous even in reduced strength. The Edison current is perfectly safe.

Very truly yours, G. BETTON MASSEY.

1706 WALNUT STREET, PHILADELPHIA.

COMMENTS.

We are very decidedly of the opinion that it *is* presumptuous on the part of the author of the above letter to hint "that the comprehensive editorial 'we' shields, in this instance, the shoulders of a young and enthusiastic manipulator of the knife." By what right does a man presume that any responsibility lies elsewhere than with the journal which publishes an editorial? And we think it doubly presumptuous for a man to insinuate all that is implied in the expressions, "young and enthusiastic manipulator of the knife," "ultra-surgical extremist," etc., especially when the suspect stands in precisely the same position as his opponent. Does Dr. Massey forget that the difference in age between himself and his suspected[1] critic may be counted by weeks? Is it possible for him to have overlooked the fact that he is not only a young man himself in years, but that he has had only about a year and a half or two years' experience in gynecology? Does he think a man can have obtained, in so short a time, so thorough a knowledge of gynecology as to write a book on the subject, and presume to speak with the authority he does thereon? And, these things being true, can he be surprised that there is some hesitation in accepting his extreme opinion based on such a *very limited experience?*

We hardly see how we can be justly accused of being an "opponent of all electrical applications in gynecology," when such an expression as "important branch of gynecological work" is used in this connection, and as we have elsewhere spoken of the valuable results obtained by this remedy. Nor do we see the slightest inconsistency in the paragraph quoted by Dr. Massey and the final paragraph in our editorial review. Our meaning is clear, and it is incomprehensible how it could be misunderstood. If for the words "*should be*" Dr. M. will substitute "*ought to have been*," he will surely comprehend the full import of the sentence.

We see no dishonesty in Dr. M.'s having picked his cases to illustrate his points; he seems to be hypersensitive on this subject. It is customary, as well as perfectly right and proper, for an author to select the most illustrative cases possible in support of any given assertion. That these cases were picked is quite evident, but it had been quite as well had some failures been given; not that we do not think many of them were failures, but they are not given as such.

We have little additional to say about the use of the meter. Our quotations from Dr. M.'s book show conclusively that he uses the patient's sensations as his guide. He now says he "has seen the same evidences of pain from fifteen milliampères in one woman as were produced by 350 in another," and offers this as proof that the meter is an absolute necessity. Where the pain is all the woman can bear with fifteen milliampères, he must stop, whether he looks at his meter and sees the register or not. The pain is what makes him stop, not the meter. And so it is with the 350. He stops when the pain is reached, not because the meter registers so many milliampères. He asks, "Would the critic banish scales from the drug store?" etc. "Is there no use in knowing how much quinine or opium is given?" etc. If we are allowing the patient herself to administer the drugs, then it becomes absolutely necessary to know how much they are taking. But here, in applying electricity, the physician himself, with

1. Unless we are sadly at fault in our guessing.

his own hands, makes the application. Who of us have not pushed quinine to cinchonism, or opium to narcotism, without caring one jot how much it took, guided only by the results? Have we not done it frequently, and with perfect confidence and safety? And so it is with electricity. He who would give it arbitrarily, in such-and-such doses, irrespective of symptoms, would be an unsafe man. Again, is it necessary to be perfectly familiar with all the technicalities of currents and their powers, as assumed by Dr. M.? By no means. We all constantly violate such rules daily. Who knows anything about antipyrin, and who does not constantly use it with the best results, and with perfect safety? And so it is with many other therapeutic agents. Such knowledge is desirable, but not necessary.

In regard to interpolar action, we must still differ with Dr. M. We do not care one whit for the opinion of any man unless it is well backed by proof. Now, what proof does the author offer us of this much-disputed point?—for to assume that it is settled is absurd. He simply quotes from, and refers the reader to, the experiments of Inglis Parsons (*British Gyn. Journal*, May, 1888, p. 78). To show how Parsons is made to prove something which he himself says he does not prove, and which he also says is only "conceivable," the readers of the JOURNAL are referred to the original article of Inglis Parsons, and also to a review of Dr. Massey's book, published in the *Annals of Gynecology* for June, 1889, p. 416, where these experiments are gone into in detail, and the misconstruction clearly shown.

Dr. M. upholds his opinion as to the propriety of treating extra-uterine pregnancy, first, by electricity, and then to perform laparatomy, and quotes Gaillard Thomas in support of this view. He also still holds that if a mistake in diagnosis be made, no harm will be done the mother. Harm can readily be done the mother, and the reader has only to refer to page 193 of the author's book to see that in the case of papillomatous cyst he himself had to stop the applications in order to save the patient's life. It is unnecessary to here produce further proof of this fact, although it would be easy to show an abundant quantity, and, no doubt, Dr. M. could tell many such experiences himself. The time when the *ipse dixit* of any man can be set up as law has passed, and with all due respect to the experience of Gaillard Thomas, we *do not* agree with him in his conclusions. What is of importance to us is: On what data are his conclusions based? This consists, principally, of two papers read before the American Gynecological Society in 1882 and 1884. He here reports twenty-seven cases. Any one reading this report will be struck by the frequency with which his diagnosis was disputed by others; how often he was doubtful of his own diagnosis; even where the diagnosis was made, how very seldom a *post mortem* was made to prove it; how often a rupture had taken place before electricity was applied or even the diagnosis made; on what slight signs the diagnosis was arrived at; in fact, the whole chain of evidence presented is so poor that it is by no means satisfactory or convincing. Advanced by a less eminent authority, the evidence presented to back his opinions would not even be seriously considered. It proves not a single conclusion to which the author has arrived. On the contrary, daily experience and well-authenticated results are rapidly bringing the profession to an opposite opinion. To say that it is proper to first kill the fetus and then remove the dèbris, is to acknowledge one's self incompetent to safely deal with a tubal pregnancy primarily by surgical means. There are few competent abdominal surgeons who are forced to make such a humiliating admission. Even with all the evidence in favor of the electrical treatment before him, an equally eminent gynecologist (Goodell) pronounces for the knife.—*The Editors.*

---

How to Clean Hypodermic Syringes.—Syringes, whose canals have become obstructed so that a fine wire cannot be drawn through, are cleaned by holding them for a moment over a flame; the foreign substance is thus quickly destroyed and driven off. If a wire has been rusted into the needle, it should be dipped in oil before holding over the flame. To remove the rust from the interior of the canula, it is well to pass oil through the canula, then heating it; then rinse it out with alcohol. The needle is then ready for use.—*Deutsch. Med. Wochenschr., 1889.*

---

Cystitis in Women.—Dr. Thomas Moore Madden, of Dublin, treats severe cystitis in women by dilatation of the urethra. This permits a continuous outflow of the secretions. In many cases, this treatment, together with mild washing of the bladder, will effect a speedy cure. If not, the fundus and neck of the bladder should be wiped off with a tampon of cotton soaked in carbolized glycerine and inserted through the dilated urethra. The use of cocaine will relieve all pain caused by this operation.—*Medical and Surgical Reporter.*

